# Comparative efficacy of repetitive peripheral magnetic stimulation and transcutaneous electrical nerve stimulation in upper trapezius myofascial pain syndrome

**DOI:** 10.55730/1300-0144.6178

**Published:** 2026-02-09

**Authors:** Shahriyar KALBİYEV, Şehim KUTLAY, Atilla Halil ELHAN, Yeşim KURTAİŞ AYTÜR, Aysun GENÇ

**Affiliations:** 1 Department of Physical Medicine and Rehabilitation, Faculty of Medicine, Ankara University, Ankara, Turkiye; 2 Department of Biostatistics, Faculty of Medicine, Ankara University, Ankara, Turkiye

**Keywords:** Myofascial pain syndrome, repetitive peripheral magnetic stimulation, TENS, ultrasound elastography, trigger points

## Abstract

**Background/aim:**

In this prospective, randomized, single-blind study, the effects of repetitive peripheral magnetic stimulation (rPMS) and transcutaneous electrical nerve stimulation (TENS) on pain intensity, functional status, and tissue elasticity were evaluated in patients with upper trapezius myofascial pain syndrome (MPS).

**Materials and methods:**

A total of 75 female patients were randomly allocated to three groups: rPMS, TENS, and sham rPMS. All participants were provided with an exercise program consisting of cervical range-of-motion (ROM), stretching, and strengthening exercises. Outcome measures included pain intensity assessed using the visual analog scale, pressure pain threshold (PPT), the Neck Disability Index, cervical ROM, SF-36 subscale scores, and ultrasound shear-wave elastography. All data were collected and compared before treatment, immediately after treatment, and 1 month after treatment.

**Results:**

Both rPMS and TENS significantly reduced pain intensity, increased PPT, and improved functional outcomes compared with the sham rPMS group. However, rPMS was associated with a greater increase in PPT compared with TENS (p = 0.013). A significant improvement in ROM was observed across all three groups for all neck positions, except for extension in the sham rPMS group. The rPMS group showed significant improvements in four SF-36 subscales compared with the sham rPMS group, whereas the TENS group showed no significant differences. Furthermore, improvements in physical functioning (p = 0.011) and mental health (p = 0.007) scores were significantly greater in the rPMS group than in the TENS group. A significant increase in muscle elasticity was observed only in the TENS group; no significant change was observed in the rPMS or sham rPMS groups.

**Conclusion:**

The findings may suggest that rPMS may be a promising noninvasive modality for the management of MPS. It may be used to reduce pain and improve functional outcomes in patients with MPS. Elastography findings may indicate that neurophysiological mechanisms contribute substantially to pain and dysfunction; however, mechanical stiffness cannot be excluded as a contributing factor. Longer follow-up studies are needed to determine the effectiveness of rPMS treatment protocols.

## 1. Introduction

Myofascial pain syndrome (MPS) is a common regional pain disorder characterized by the presence of trigger points, taut bands within skeletal muscle, and referred pain. As a leading cause of noninflammatory chronic pain, MPS often arises from sustained muscle hyperactivity or from acute or chronic musculoskeletal overload. Etiologically, MPS has been associated with repetitive microtrauma, postural strain, or psychological stress, which may lead to sustained acetylcholine release at the neuromuscular junction and a subsequent energy crisis within muscle fibers [1,2]. While MPS is distinct from inflammatory myopathies, its burden on quality of life and healthcare systems underscores the need for effective therapies. Despite multimodal therapeutic approaches, including exercise, patient education, pharmacotherapy, physical therapy, and electrotherapy, no universally accepted gold-standard treatment has been established.

Repetitive peripheral magnetic stimulation (rPMS) is an emerging noninvasive technique that produces analgesic effects through magnetic field–induced electrical pulses and is thought to target deep tissues by stimulating muscle mechanoreceptors and sensorimotor nerve fibers. This modality employs high-frequency magnetic pulses (≥1 Hz) to generate electrical currents in deep tissues without causing cutaneous discomfort. Its therapeutic effects are attributed to analgesic properties, muscle relaxation, and improved perfusion [3]. Transcutaneous electrical nerve stimulation (TENS), another physical therapy modality widely used for pain management, involves the delivery of low-frequency alternating electrical currents through superficial cutaneous electrodes and is believed to exert analgesic effects through the gate control theory, regional vasodilation, increased endogenous opioid release, and sympathetic blockade [4]. Although TENS is widely used in the management of myofascial pain, rPMS has emerged as a potential alternative because of its deeper tissue penetration, preferential activation of proprioceptive afferents, and minimal cutaneous stimulation [3]. Given the limited comparative evidence between these two methods and the absence of studies integrating objective biomechanical evaluation, a direct comparison of rPMS and TENS is necessary to clarify their relative clinical efficacy.

This study aimed to compare the effectiveness of rPMS and TENS in improving clinical symptoms and functional outcomes in patients with upper trapezius MPS and to assess mechanical changes in myofascial trigger points using ultrasound elastography.

## 2. Materials and methods

This study was designed as a prospective, randomized, single-blind, assessor-blinded, controlled clinical trial. The study protocol was approved by the Ethical Committee of Ankara University School of Medicine (approval no: I02-65-23). The research was carried out in compliance with the Declaration of Helsinki and informed consent was obtained from all participants. A total of 176 female patients presenting to the physical medicine and rehabilitation outpatient clinic and exhibiting at least one active trigger point in the upper trapezius muscle were screened for eligibility according to the predefined exclusion criteria. To ensure sample homogeneity and reduce sex-dependent variability in muscle elastic properties assessed by shear-wave elastography (SWE), only female patients were included. Among the 75 randomized patients, 23 presented with multiple trigger points in the upper trapezius muscle; in such cases, the most painful trigger point was selected for evaluation. Exclusion criteria were as follows: symptom duration greater than 1 month, any treatment for myofascial pain within the last 3 months, presence of kyphosis or scoliosis, cervical disc herniation, concomitant radiculopathy or myelopathy, presence of a pacemaker, history of neck or shoulder surgery, diagnosis of malignancy, fibromyalgia, and uncontrolled hypertension. After the exclusion of 101 patients during screening, the remaining 75 eligible patients were randomized using Random Allocation Software (version 1.0.0; Isfahan University of Medical Sciences, Isfahan, Iran) and allocated in a 1:1:1 ratio to the rPMS, TENS, or sham rPMS groups. Block randomization was used, with a block size of three. In both the TENS and sham rPMS groups, four patients discontinued the intervention due to incompatibility and exercise intolerance and were excluded from the final analysis. The study flowchart is depicted in Figure.

All participants across the three groups were provided with an exercise program that included cervical range-of-motion (ROM) exercises, stretching exercises, and strengthening exercises. The rPMS device (BTL-6000 Super Inductive System; BTL Industries Ltd., Stevenage, UK) was administered once daily for 2 weeks (five sessions per week), for a total of 10 sessions. Each session lasted 10 min and was applied to the painful trapezius muscle while the participant was seated. The manufacturer’s “1001 Muscle Relaxation” protocol was used. This protocol consisted of seven segments, each characterized by modulated current and frequency, with a total duration of 10 min. The pulse waveforms were sinusoidal, biphasic (alternating), and trapezoidal, with a frequency range of 1–150 Hz and a pulse duration of 280 μs. The stimulation intensity was adjusted for each patient to ensure painless stimulation, based on the device’s maximum output. TENS was administered for 2 weeks (one session per day, five sessions per week), for a total of 10 sessions. Each session lasted 20 min and was applied to the painful trigger points of the trapezius muscle while the participant was seated. Conventional TENS (BioMed 2000 TENS Unit; BioMedical Life Systems Inc., Carlsbad, California, USA) was applied at a frequency of 50–100 Hz and a pulse duration of 100–200 μs for 20 min. The stimulation intensity was adjusted for each patient to ensure that stimulation was painless. In the sham rPMS group, the rPMS device was not activated; however, the probe was positioned identically, and a prerecorded device sound was played for 10 min. Adverse events were actively monitored throughout the intervention period. At each treatment session, participants were asked about any discomfort or adverse effects, and events such as local pain, skin irritation, dizziness, or other unexpected symptoms were documented. No serious adverse events occurred in either group.

### 2.1. Outcome measures

The 100-mm visual analog scale (VAS) was used to assess neck pain intensity and response to treatment. Active trigger points were identified by palpation of the cervical and back regions. The number of painful trigger points was recorded before and after treatment, and the most painful trigger point was marked with a pen. A handheld digital pressure algometer (J-Tech Commander Algometer; JTECH Medical, Midvale, USA) was used to measure PPT; measurements were obtained three times at 60 s intervals over the most painful trigger point, and the arithmetic mean was recorded in kg/cm^2^. Cervical spine ROM was measured using a goniometer for flexion, extension, bilateral lateral flexion, and bilateral rotation. Disability was assessed using the validated Turkish version of the 20-item Neck Disability Index (NDI; 0%–100%) [5]. The Turkish version of the SF-36 was used to assess quality of life [6]. Furthermore, the effects of the treatment modalities on the mechanical properties of active trigger points were quantitatively assessed using ultrasound shear-wave elastography (SWE). Each participant was assessed using the Siemens ACUSON S2000 Ultrasound Virtual Touch Tissue Quantification system (Siemens Healthineers, Erlangen, Germany) while seated, with the shoulders and neck exposed and the shoulder positioned at rest and at 90° of abduction. Images were acquired using a 9-MHz linear-array transducer for SWE. The probe was placed perpendicular to the upper trapezius muscle without applying direct pressure to the skin. First, a quality map was obtained within the predefined region of interest to ensure optimal shear-wave signal quality. When the quality map was deemed satisfactory, the ultrasound display was switched to a static color map of the area of interest. Subsequently, measurements were obtained from six points within the quality-approved area, and the mean value was used for analysis. Tissue elasticity was quantified in m/s. All measurements were performed by a single investigator with 10 years of experience in elastography (A.G.). All data were collected before treatment, immediately after treatment, and 1 month after treatment.

### 2.2. Study design and sample size calculation

The study was conducted using a single-blind, assessor-blinded design: participants were blinded to treatment allocation; outcome assessments were performed by an independent physician who was unaware of group assignments, and treatment administration was performed by separate clinicians.

Statistical power for the Kruskal–Wallis test was estimated using a Monte Carlo simulation approach appropriate for rank-based nonparametric inference. Assuming three groups with equal sample sizes (n = 25 per group), 10,000 simulated datasets were generated under the alternative hypothesis, calibrated to a Kruskal–Wallis epsilon-squared (ɛ^2^) effect size of 0.20. Each dataset was analyzed using a two-sided Kruskal–Wallis test with a significance level of α = 0.05. Statistical power was defined as the proportion of simulations yielding p < 0.05. Under these assumptions, the estimated statistical power was approximately 93.5%; however, this estimate was based on equal group sizes and may not fully reflect the reduced final sample following attrition.

### 2.3. Statistical analysis

Descriptive statistics are presented as percentages for categorical variables; means and standard deviations for normally distributed continuous variables; and medians with 25th and 75th percentiles for ordinal and nonnormally distributed continuous variables. Differences in proportions between groups were compared using the chi-square test or Fisher’s exact test, as appropriate. Differences among the three groups in ordinal or nonnormally distributed continuous variables were evaluated using the Kruskal–Wallis test. When the Kruskal–Wallis test yielded a statistically significant result, Dunn’s test was used to identify between-group differences. Friedman’s two-way analysis of variance by ranks was used to evaluate within-group differences over time. When the Friedman test yielded a statistically significant result, Dunn’s test was used for post hoc pairwise comparisons. The Bonferroni correction was applied to all multiple comparisons to control the Type I error rate. All statistical analyses were conducted using IBM SPSS Statistics (version 30.0; IBM Corp., Armonk, NY, USA) and a p < 0.05 was considered statistically significant.

## 3. Results

Demographic characteristics of the three groups are presented in Table 1. No adverse events were reported in any group during the study period. Analysis of sociodemographic characteristics revealed no statistically significant differences among the groups in age, body mass index (BMI), education level, marital status, occupation, comorbidities, medication history, treated side, or number of trigger points (p > 0.05). The mean age was 38.08 ± 8.82 years in the rPMS group, 39.81 ± 8.75 years in the TENS group, and 38.43 ± 8.84 years in the sham rPMS group. There were no significant differences between the groups in pretreatment pain VAS scores (p = 0.714), pain threshold (p = 0.558), and NDI scores (p = 0.463). The severity of pain decreased significantly with treatment in all three groups (p < 0.001). Although reductions in VAS scores in the rPMS and TENS groups were greater than those observed in the sham rPMS group, no statistically significant difference was detected between the rPMS and TENS groups. Analysis of changes in pain-threshold scores demonstrated significant increases in the rPMS and TENS groups (p < 0.001), whereas no significant change was observed in the sham rPMS group (p = 0.170). When changes in pain-threshold scores from baseline to immediately after treatment were compared, significant differences were observed in the rPMS (p < 0.001) and TENS (p = 0.004) groups compared with the sham rPMS group; no significant difference was detected between the rPMS and TENS groups (p = 0.091). However, when baseline values were analyzed together with those observed 1 month after treatment, the rPMS group demonstrated a significantly greater improvement in pain threshold than the TENS (p = 0.013) and sham rPMS (p < 0.001) groups. No significant difference was detected between the TENS and sham rPMS groups (p = 0.185). When analyzing within-group changes in NDI scores, all groups showed significant decreases across all time periods (p < 0.001). When changes in NDI scores from baseline to immediately after treatment were compared, significant differences were observed between the rPMS and sham rPMS groups (p < 0.001) and between the TENS and sham rPMS groups (p < 0.001); no significant difference was detected between the rPMS and TENS groups (p = 0.999). However, when scores at baseline were compared with those obtained 1 month after treatment, the previously observed statistical significance between the TENS and sham rPMS groups was no longer present (p = 0.208; Table 2).

Cervical ROM was assessed in flexion, extension, bilateral rotation, and bilateral lateral bending. Regarding baseline group comparability, flexion (p = 0.013) and extension (p = 0.041) ROM values were significantly lower in the sham rPMS group than in the other groups. When evaluating the response to treatment within groups, ROM showed a statistically significant increase in all positions across all groups, except for extension ROM in the sham rPMS group (p = 0.195). No significant differences were observed among the groups when changes in cervical flexion, extension, bilateral rotation, and bilateral lateral bending were compared from baseline to immediately after treatment and from baseline to 1 month after treatment. A significant difference was observed between the rPMS and sham rPMS groups only with respect to the increase in cervical right-rotation ROM from baseline to immediately after treatment (p = 0.024) (Table 3).

The SF-36 scores are presented in Table 4. When comparing pretreatment scores between groups, only the general health subscore was significantly higher in the TENS group (p = 0.024). Within-group analyses over time demonstrated significant improvement in seven SF-36 subscales in the rPMS group and six subscales in the TENS group, whereas only two subscales improved in the sham rPMS group. When SF-36 score changes were compared between treatment groups, significant improvements were observed in the rPMS group compared with the sham rPMS group in the physical functioning, role physical, social functioning, and general health subscales, whereas no statistically significant differences were detected between the TENS and sham rPMS groups for any SF-36 parameters. In addition, improvements in physical functioning (p = 0.011) and mental health (p = 0.007) scores were significantly greater in the rPMS group than in the TENS group.

Baseline SWE values of the trapezius muscle, both at rest and at 90° of abduction, were significantly lower in the sham rPMS group, indicating baseline between-group differences. In within-group analyses, a significant increase in elasticity in the shoulder-rest position was detected only in the TENS group (p = 0.002), whereas no significant change in muscle elasticity was observed in the rPMS and sham rPMS groups. When changes in elasticity were compared between groups, no significant differences were observed between the rPMS and sham rPMS groups or between the rPMS and TENS groups; however, the increase in elasticity from baseline to immediately after treatment in the TENS group was significantly greater than that observed in the sham rPMS group (p = 0.029) (Table 5).

## 4. Discussion

To the best of our knowledge, this is the first study to compare rPMS and TENS for the management of upper trapezius myofascial pain and to incorporate elastography for the evaluation of biomechanical changes. Treatment with rPMS was associated with meaningful clinical benefits, with higher pain thresholds and improved SF-36 physical and mental health scores observed 1 month after treatment compared with TENS. However, it did not result in greater improvements in ROM or in trigger point elasticity.

Magnetic therapy has been used for several decades to treat pain in various musculoskeletal conditions; in particular, pulsed electromagnetic fields have been reported to be effective in the management of knee and cervical spondylosis and rotator cuff tendinitis [7–9]. It should be noted that the magnetic stimulation procedure used in this study differs from conventional magnetic therapy, as rPMS can be delivered at substantially higher intensities than those achievable with conventional systems [10]. The potential benefit of using a high-intensity magnetic field lies in its ability to penetrate and stimulate deep tissues, which may contribute to modulation of abnormal neurotransmission associated with chronic muscle pain. Furthermore, whereas conventional magnetic therapy is typically applied to larger anatomical regions (e.g., trunk or limbs), rPMS may be used to target more localized pain conditions, such as myofascial trigger points [11–13].

Noninvasive peripheral magnetic stimulation involves the use of external devices to induce muscle contractions and sensory afferent activity through depolarization of peripheral neural structures. Similar to TENS, both modalities are thought to activate underlying tissues through voltage gradients and ion fluxes, with proposed effects ranging from local neuromuscular modulation and synaptic facilitation within spinal circuits to alterations in frontoparietal and corticospinal excitability [14]. This modality is generally considered a relatively painless technique that may preferentially engage proprioceptive afferents rather than cutaneous fibers [15]. However, its clinical uptake is limited by insufficient high-quality evidence, unclear stimulation parameters, and ongoing debate regarding whether such proprioceptive selectivity occurs and confers therapeutic benefit in pathological conditions [16]. Smania et al. conducted a comparative analysis of the short-, medium-, and long-term effects (3 months after treatment completion) of rPMS versus transcutaneous electrical nerve stimulation (TENS) in patients with myofascial pain [13]. In that study, significant improvements were observed in the rPMS group in neck pain intensity (visual analog scale), disability, algometric measurements, trigger point characteristics, and contralateral cervical rotation, whereas significant improvements were also reported in the TENS group in similar outcome measures, with the exception of algometry. No significant improvement was observed in the TENS group at the 3 month follow-up, leading the authors to suggest that peripheral rPMS may be more effective than TENS in the treatment of myofascial pain. In the present study, patients with symptom duration of less than 1 month were evaluated; therefore, the analysis focused on early and midterm outcomes. Consistent with these findings, a significant improvement in algometric measurements was observed in favor of rPMS at 1 month. Improvements in pain intensity and NDI scores were significantly greater in the rPMS and TENS groups than in the sham rPMS group; however, this difference was no longer observed in the TENS group at the 1-month follow-up. Unlike the findings reported by Smania et al., no significant differences were found in these parameters between rPMS and TENS.

In addition to the functional outcomes evaluated in this study, ultrasound elastography was utilized to directly and quantitatively assess muscle stiffness and tissue mechanical properties. Previous studies have reported that muscles located in trigger point regions exhibit increased resting stiffness [17,18]. This muscle stiffness can be quantified using SWE. Because SWE may detect microenvironmental changes earlier than conventional ultrasound and provides quantitative information regarding tissue stiffness, its clinical use has increased [18]. However, in contrast to these findings, Kobylarz et al. reported no significant differences in muscle stiffness between latent trigger points and surrounding control tissues during SWE assessments [19]. They suggested that the hypothesis proposing a positively association between muscle stiffness and mechanosensitivity, as well as the role of stiffness in trigger point diagnosis, was not supported by their findings. In the present study, elastography measurements of the upper trapezius muscle were obtained from six regions of interest in patients with symptom duration of less than 1 month. The aim of the present study was not to compare trigger points with surrounding muscle tissue, but rather to evaluate the biomechanical effects of rPMS and TENS on muscle tissue and to determine whether these biomechanical findings parallel functional outcomes. Although the rPMS group demonstrated limited advantages over the TENS group in certain functional outcomes, a significant increase in muscle elasticity was observed only in the TENS group, and elasticity responses did not differ significantly between the rPMS and sham rPMS groups. These findings further support the possibility that pain and dysfunction in MPS may be more closely associated with neurophysiological processes than with mechanical stiffness, as discussed by Kobylarz et al. [19].

Vearasilp and Sukareechai applied rPMS to relieve pain following dry needling of active myofascial trigger points in the upper trapezius muscle and reported significant improvements in pressure pain threshold and pain intensity compared with a placebo group [20]. The authors concluded that rPMS was effective in reducing post–dry needling pain in patients with MPS and suggested that it may enhance patient comfort and recovery in clinical practice. Wu et al., in a perspective article [21], discussed the potential synergistic effects of ultrasound-guided multifidus muscle injections combined with rPMS therapy in patients with chronic myofascial or lumbar pain. They suggested that this combined treatment may provide improved pain control and functional outcomes in patients with chronic low back pain and could represent a promising multimodal strategy.

The literature reports considerable variability in the parameters used in rPMS applications, including intensity, frequency, treatment duration, pulse rate, pulse duration, and number of sessions; a standardized treatment protocol has not yet been established. Existing studies have applied protocols consisting of 1–20 sessions and 500–5000 pulses delivered over 3–20 min, using circular or figure-of-eight coils, biphasic currents, pulse durations of 5–100 μs, frequencies of 1–100 Hz, and stimulation intensities of 42%–45% of the device’s maximum output [22–24]. In the present study, 10 rPMS sessions, each consisting of seven 10 min segments, were administered to trigger points in the upper trapezius muscles using a round coil and sinusoidal biphasic (alternating) and trapezoidal currents, with a pulse duration of 280 μs, a frequency range of 1–150 Hz, painless stimulation, and a maximum stimulation intensity of 25%. Therefore, direct one-to-one comparisons between studies may not be appropriate because of substantial differences in treatment protocols.

The limitations of the present study include the absence of a comparison between the viscoelastic properties of the upper trapezius muscle and those of a healthy control population. Furthermore, conducting the final evaluation only 1 month after treatment does not provide information on the long-term effects of rPMS. Although an a priori sample size calculation was performed, participant attrition reduced the final analyzed sample. Among the initially screened patients, exclusions were applied strictly according to predefined eligibility criteria, and eight participants discontinued participation after one or two sessions and were lost to follow-up; this attrition may limit the generalizability of the findings. A post hoc sensitivity analysis based on the primary outcome suggested high statistical power to detect the observed between-group differences; however, post hoc power estimates should be interpreted with caution because of their inherent methodological limitations. Because outcome data were unavailable for participants who discontinued early, an intention-to-treat analysis could not be performed; therefore, a per-protocol analysis was conducted. This limitation should be considered when interpreting the results. Finally, only female patients were included, which further limits the generalizability of the results.

In conclusion, rPMS appears to be a noninvasive, easily applied, and well-tolerated intervention with no serious adverse effects observed in this study. It may be considered a potentially effective physical therapy modality for reducing pain and improving functional outcomes in patients with MPS. The lack of a consistent association between functional improvement and elastography findings may indicate that pain and dysfunction in MPS are influenced more by neurophysiological mechanisms than by mechanical tissue stiffness. However, future studies with longer follow-up intervals are required to optimize rPMS treatment protocols.

## Figures and Tables

**Figure:**
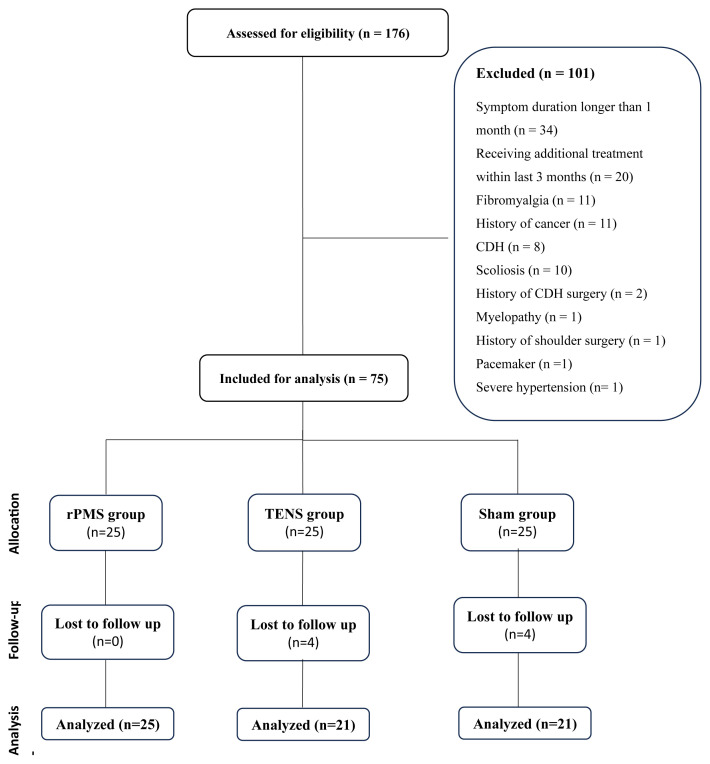
Flow diagram of the study.

**Table 1:** Demographic and clinical characteristics of the three groups.

Variable	rPMS group	TENS group	Sham rPMS group	p
	n = 25	n = 21	n = 21	

Age (years), mean ± SD	38.08 ± 8.82	39.81 ± 8.75	38.43 ± 8.84	0.464

BMI (kg/m^2^), mean ± SD	25.61 ± 4.06	24.24 ± 3.22	26.35 ± 5.11	0.521

Educational level, n (%)				0.662
Primary-secondary school	15 (60%)	13 (62%)	14 (67%)	
High school	8 (32%)	5 (38%)	7 (33%)	
University	2 (8%)	0 (0%)	0 (0%)	

Marital status, n (%)				0.749
Single	6 (25%)	7 (33%)	5 (24%)	
Married	18 (75%)	14 (67%)	16 (76%)	

Employment status, n (%)				0.095
Homemaker	13 (52%)	11 (52.4%)	12 (57.1%)	
Manual laborer	1 (4%)	3 (14.3%)	6 (28.6%)	
Office worker	11 (44%)	7 (33.3%)	9 (14.3%)	

Medication history, n (%)				0.229
None	6 (24%)	13 (61.9%)	9 (45%)	
Paracetamol	4 (16%)	3 (14.3%)	3 (15%)	
NSAIDs	9 (36%)	2 (9.5%)	4 (20%)	
Muscle relaxants	6 (24%)	3 (14.3%)	4 (20%)	

Treatment area, n (%)				0.104
Left	14 (56%)	12 (57.1%)	6 (28.6%)	
Right	11 (44%)	9 (42.9%)	15 (71.4%)	

The number of trigger points, median (min–max)	1 (1–3)	1 (1–2)	1 (1–3)	0.429

rPMS: repetitive peripheral magnetic stimulation; TENS: transcutaneous electrical nerve stimulation; BMI: body mass index; NSAID: nonsteroidal antiinflammatory drug.

**Table 2:** Intergroup comparison of treatment response in patients with myofascial pain syndrome.

Variable														
**VAS (0–10 cm)**		**T1**	**T2**	**T3**	**p1**	**p2**	**pT1**–**T2**	**pT1**–**T3**	**p3**	**p4**	**p5**	**p6**	**p7**	**p8**
	**rPMS group**	8 (8–10)	3 (2–4)	4 (2–5)	0.714	**<0.001**	**<0.001**	**<0.001**	0.105	0.441	**<0.001**	**<0.001**	**<0.001**	**0.002**
	**TENS group**	8 (7–10)	4 (3–5)	4 (3–6)		**<0.001**	**<0.001**	**<0.001**						
	**Sham rPMS group**	8 (7–9)	6 (6–8)	6 (5–8)		**<0.001**	**0.001**	**0.004**						
**Pain threshold, kg/cm** ^2^	**rPMS group**	1 (0.6–0.8)	2.5 (1.7–2.8)	2.3 (2–2.5)	0.558	**<0.001**	**<0.001**	**<0.001**	0.091	**0.013**	**<0.001**	**<0.001**	**0.004**	0.185
	**TENS group**	1 (0.8–1.2)	1.7 (1.4–1.9)	1.5 (1.1–1.8)		**<0.001**	**<0.001**	**0.008**						
	**Sham rPMS group**	1 (0.6–1.5)	1 (0.9–1.5)	1.1 (0.9–1.4)		0.170								
**NDI (0%–100%)**	**rPMS group**	29 (21–34)	18 (14–20)	18 (13–22)	0.463	**<0.001**	**<0.001**	**<0.001**	0.999	0.237	**<0.001**	**0.001**	**<0.001**	0.208
	**TENS group**	23 (21–33)	17 (14–22)	20 (18–25)		**<0.001**	**<0.001**	**0.008**						
	**Sham rPMS group**	24 (22–27)	21 (18–25)	21 (19–25)		**<0.001**	**<0.001**	**<0.001**						

Data are presented as median (25th–75th percentiles). rPMS: repetitive peripheral magnetic stimulation; TENS: transcutaneous electrical nerve stimulation; NDI: Neck Disability Index. T1: baseline; T2: immediately after treatment; T3: 1 month after treatment. p1: comparison of baseline values among groups; p2: comparison of change over time within the group; p3: comparison of the change from T1 to T2 between the rPMS and TENS groups; p4: comparison of the change from T1 to T3 between the rPMS and TENS groups; p5: comparison of the change from T1 to T2 between the rPMS and sham rPMS groups; p6: comparison of the change from T1 to T3 between the rPMS and sham rPMS groups; p7: comparison of the change from T1 to T2 between the TENS and sham rPMS groups; p8: comparison of the change from T1 to T3 between the TENS and sham rPMS groups. p-values less than 0.05 are indicated in bold.

**Table 3:** Intergroup comparison of cervical range of motion (degrees).

**Flexion**		**T1**	**T2**	**T3**	**p1**	**p2**	**pT1**–**T2**	**pT1**–**T3**	**p3**	**p4**	**p5**	**p6**	**p7**	**p8**
	**rPMS group**	45 (42–47)	46 (44–48)	47 (45–48)	**0.013**	**<0.001**	**0.004**	**<0.001**	0.989	0.969	0.686	0.328	0.624	0.488
	**TENS group**	45 (44–46)	46 (45–47)	46 (45–48)		**<0.001**	**0.004**	**<0.001**						
	**Sham rPMS group**	41 (40–44)	43 (41–45)	42 (42–45)		**<0.001**	**0.002**	**0.001**						
**Extension**	**rPMS group**	54 (50–55)	55 (51–56)	54 (50–56)	**0.041**	**0.033**	0.071	0.774	0.993	0.307	0.816	0.905	0.770	0.571
	**TENS group**	52 (51–55)	54 (52–55)	55 (52–56)		**0.017**	0.840	0.092						
	**Sham rPMS group**	50 (49–52)	50 (50–52)	51 (50–52)		0.195								
**Right rotation**	**rPMS group**	67 (63–70)	71 (68–72)	70 (68–72)	0.904	**<0.001**	**0.001**	**0.007**	0.671	0.876	**0.024**	0.323	0.226	0.630
	**TENS group**	65 (62–70)	70 (65–72)	70 (67–72)		**<0.001**	**0.001**	**0.001**						
	**Sham rPMS group**	66 (65–68)	68 (66–70)	69 (66–70)		**0.001**	**0.026**	**0.005**						
**Left rotation**	**rPMS group**	67 (65–71)	70 (67–73)	70 (70–72)	0.499	**0.007**	**0.017**	0.102	0.932	0.999	0.944	0.814	0.999	0.814
	**TENS group**	65 (60–71)	68 (63–71)	67 (65–71)		**0.002**	0.062	**0.008**						
	**Sham rPMS group**	66 (63–70)	68 (65–70)	70 (66–70)		**<0.001**	0.161	**0.002**						
**Right lateral bending**	**rPMS group**	38 (34–40)	39 (36–41)	40 (36–41)	0.312	**<0.001**	**0.001**	**0.001**	0.813	0.854	0.319	0.650	0.701	0.365
	**TENS group**	35 (34–40)	38 (36–40)	40 (36–41)		**<0.001**	**<0.001**	**0.041**						
	**Sham rPMS group**	38 (37–40)	40 (38–41)	40 (40–40)		**0.002**	**0.026**	0.135						
**Left lateral bending**	**rPMS group**	38 (35–40)	40 (37–41)	40 (38–42)	0.318	**0.003**	**0.049**	**0.011**	0.477	0.459	0.806	0.997	0.205	0.530
	**TENS group**	36 (30–40)	40 (36–40)	39 (35–41)		**<0.001**	**0.001**	**0.008**						
	**Sham rPMS group**	37 (34–40)	40 (38–41)	40 (39–40)		**<0.001**	**<0.001**	**0.026**						

Data are presented as median (25th–75th percentiles). rPMS: repetitive peripheral magnetic stimulation; TENS: transcutaneous electrical nerve stimulation. T1: baseline; T2: immediately after treatment; T3: 1 month after treatment. p1: comparison of baseline values among groups; p2: comparison of change over time within the group; p3: comparison of the change from T1 to T2 between the rPMS and TENS groups; p4: comparison of the change from T1 to T3 between the rPMS and TENS groups; p5: comparison of the change from T1 to T2 between the rPMS and sham rPMS groups; p6: comparison of the change from T1 to T3 between the rPMS and sham rPMS groups; p7: comparison of the change from T1 to T2 between the TENS and sham rPMS groups; p8: comparison of the change from T1 to T3 between the TENS and sham rPMS groups. p-values less than 0.05 are indicated in bold.

**Table 4:** SF-36 health survey scores.

**Physical functioning**		**T1**	**T2**	**T3**	**p1**	**p2**	**p2 (T1**–**T2)**	**p2 (T1**–**T3)**	**p3**	**p4**	**p5**	**p6**	**p7**	**p8**
	**rPMS group**	35 (25–55)	70 (55–80)	70 (55–80)	0.452	**<0.001**	**0.001**	**<0.001**	0.311	**0.011**	**0.001**	**<0.001**	0.134	0.946
	**TENS group**	50 (30–55)	65 (50–85)	60 (45–70)		**<0.001**	**<0.001**	**0.017**						
	**Sham rPMS group**	45 (35–50)	52.5 (37.5–62.5)	50 (40–70)		0.125	0.309	0.309						
**Role physical**	**rPMS group**	0 (0–25)	50 (25–75)	50 (25–75)	0.692	**<0.001**	**0.003**	**0.003**	0.863	0.624	**0.006**	**0.032**	0.156	0.638
	**TENS group**	25 (0–25)	40 (15–75)	40 (25–60)		**0.002**	**0.022**	**0.011**						
	**Sham rPMS group**	0 (0–50)	0 (0–75)	20 (0–70)		0.360	1.000	1.000						
**Role emotional**	**rPMS group**	33.3 (0–33.3)	33.3 (33.3–66.7)	60 (33.3–66.7)	0.090	**<0.001**	**0.007**	**0.005**	1.000	0.217	0.096	0.194	0.577	1.000
	**TENS group**	33.3 (18–66.7)	50 (35–100)	55 (33.3–66.7)		**0.001**	**0.009**	0.152						
	**Sham rPMS group**	0 (0–33.3)	33.3 (0–45)	33.3 (0–45)		0.052	0.632	0.090						
**Vitality**	**rPMS group**	40 (20–50)	40 (25–60)	50 (30–55)	0.820	0.481	1.000	0.578	0.948	1.000	0.600	1.000	0.085	1.000
	**TENS group**	40 (30–50)	50 (45–55)	50 (40–55)		**0.002**	**0.009**	**0.014**						
	**Sham rPMS group**	30 (25–50)	35 (25–50)	45 (35–55)		0.129	1.000	0.210						
**Mental health**	**rPMS group**	48 (40–56)	56 (52–70)	55 (48–64)	0.185	**0.017**	**0.014**	0.240	**0.007**	0.085	0.138	1.000	0.952	0.177
	**TENS group**	52 (44–60)	50 (45–60)	50 (44–60)		0.619	1.000	1.000						
	**Sham rPMS group**	52 (44–60)	55 (48–68)	55 (50–65)		**0.023**	0.090	0.062						
**Social functioning**	**rPMS group**	37.5 (25–62.5)	62.5 (37.5–75)	62.5 (55–75)	0.247	**<0.001**	**0.018**	**<0.001**	1.000	0.126	0.141	**<0.001**	0.868	0.261
	**TENS group**	50 (50–62.5)	62.5 (50–70)	62.5 (50–70)		**0.003**	**0.007**	0.062						
	**Sham rPMS group**	50 (40–50)	50 (50–62.5)	50 (50–60)		0.160	0.285	0.560						
**Bodily pain**	**rPMS group**	42.5 (20–55)	55 (45–67.5)	55 (45–67.5)	0.494	**0.009**	0.095	**0.007**	0.430	0.067	0.112	0.498	1.000	1.000
	**TENS group**	32.5 (32–60)	55 (32.5–70)	55 (30–60)		0.258	0.246	1.000						
	**Sham rPMS group**	45 (32.5–55)	50 (40–55)	55 (45–60)		**0.004**	0.246	**0.009**						
**General health**	**rPMS group**	40 (30–45)	45 (35–60)	40 (35–60)	**0.024**	**0.006**	**0.012**	**0.039**	1.000	0.900	0.090	**0.049**	0.183	0.570
	**TENS group**	50 (40–60)	60 (40–70)	50 (45–65)		**0.003**	**0.005**	0.330						
	**Sham rPMS group**	45 (40–50)	45 (40–55)	45 (35–50)		0.755	1.000	1.000						

Data are presented as median (25th–75th percentiles). rPMS: repetitive peripheral magnetic stimulation; TENS: transcutaneous electrical nerve stimulation; NS: not significant. T1: baseline; T2: immediately after treatment; T3: 1 month after treatment. p1: comparison of baseline values among groups; p2: comparison of change over time within the group; p3: comparison of the change from T1 to T2 between the rPMS and TENS groups; p4: comparison of the change from T1 to T3 between the rPMS and TENS groups; p5: comparison of the change from T1 to T2 between the rPMS and sham rPMS groups; p6: comparison of the change from T1 to T3 between the rPMS and sham rPMS groups; p7: comparison of the change from T1 to T2 between the TENS and sham rPMS groups; p8: comparison of the change from T1 to T3 between the TENS and sham rPMS groups. p-values less than 0.05 are indicated in bold.

**Table 5:** Shear-wave speed values (m/s) of the trapezius muscle.

**Shoulder at rest**	**Groups**	**T1**	**T2**	**T3**	**p1**	**p2**	**p2 (T1**–**T2)**	**p2 (T1**–**T3)**	**p3**	**p4**	**p5**	**p6**	**p7**	**p8**
	**rPMS group**	2.95 (2.61–3.38)	2.78 (2.50–3.21)	2.80 (2.51–2.97)	**0.018**	0.205	0.873	0.154	0.194	0.636	1.000	1.000	**0.029**	0.133
	**TENS group**	3.2 (2.95–3.43)	2.62 (2.45–2.88)	2.65 (2.40–3.08)		**0.002**	**0.002**	**0.011**						
	**Sham rPMS group**	2.73 (2.45–2.93)	2.63 (2.35–3.19)	2.60 (2.50–2.90)		0.554	0.632	0.792						
**Shoulder at 90° abduction**	**rPMS group**	3.78 (3.3–4.27)	3.65 (3.32–4.10)	3.53 (3.35–3.72)	**0.045**	0.520	1.000	0.516	1.000	1.000	0.410	0.621	0.465	1.000
	**TENS group**	4.11 (3.28–4.69)	3.68 (3.20–4.02)	3.88 (3.44–4.29)		0.717	1.000	1.000						
	**Sham rPMS group**	3.26 (3.00–3.74)	3.58 (3.07–4.02)	3.20 (3.11–3.89)		0.867	1.000	1.000						

Data are presented as median (25th–75th percentiles). rPMS: repetitive peripheral magnetic stimulation; TENS: transcutaneous electrical nerve stimulation; NS: not significant. T1: baseline; T2: immediately after treatment; T3: 1 month after treatment. p1: comparison of baseline values among groups; p2: comparison of change over time within the group; p3: comparison of the change from T1 to T2 between the rPMS and TENS groups; p4: comparison of the change from T1 to T3 between the rPMS and TENS groups; p5: comparison of the change from T1 to T2 between the rPMS and sham rPMS groups; p6: comparison of the change from T1 to T3 between the rPMS and sham rPMS groups; p7: comparison of the change from T1 to T2 between the TENS and sham rPMS groups; p8: comparison of the change from T1 to T3 between the TENS and sham rPMS groups. p-values less than 0.05 are indicated in bold.
